# Nonrandom distribution and frequencies of genomic and EST-derived microsatellite markers in rice, wheat, and barley

**DOI:** 10.1186/1471-2164-6-23

**Published:** 2005-02-18

**Authors:** Mauricio La Rota, Ramesh V Kantety, Ju-Kyung Yu, Mark E Sorrells

**Affiliations:** 1 Department of Plant Breeding and Genetics, 240 Emerson Hall, Cornell University, Ithaca, NY, 14853, USA; 2 Department of Plant & Soil Science, 138 ARC Building, Alabama A&M University, Normal, AL, 35762, USA

## Abstract

**Background:**

Earlier comparative maps between the genomes of rice (*Oryza sativa *L.), barley (*Hordeum vulgare *L.) and wheat (*Triticum aestivum *L.) were linkage maps based on cDNA-RFLP markers. The low number of polymorphic RFLP markers has limited the development of dense genetic maps in wheat and the number of available anchor points in comparative maps. Higher density comparative maps using PCR-based anchor markers are necessary to better estimate the conservation of colinearity among cereal genomes. The purposes of this study were to characterize the proportion of transcribed DNA sequences containing simple sequence repeats (SSR or microsatellites) by length and motif for wheat, barley and rice and to determine *in-silico *rice genome locations for primer sets developed for wheat and barley Expressed Sequence Tags.

**Results:**

The proportions of SSR types (di-, tri-, tetra-, and penta-nucleotide repeats) and motifs varied with the length of the SSRs within and among the three species, with trinucleotide SSRs being the most frequent. Distributions of genomic microsatellites (gSSRs), EST-derived microsatellites (EST-SSRs), and transcribed regions in the contiguous sequence of rice chromosome 1 were highly correlated. More than 13,000 primer pairs were developed for use by the cereal research community as potential markers in wheat, barley and rice.

**Conclusion:**

Trinucleotide SSRs were the most common type in each of the species; however, the relative proportions of SSR types and motifs differed among rice, wheat, and barley. Genomic microsatellites were found to be primarily located in gene-rich regions of the rice genome. Microsatellite markers derived from the use of non-redundant EST-SSRs are an economic and efficient alternative to RFLP for comparative mapping in cereals.

## Background

The genetic maps of grass species have been constructed using a variety of marker types. Most of the older species-specific molecular maps were constructed with RFLP markers, but in recent times there has been increased utilization of PCR-based markers because of accessibility and higher throughput. Conservation of gene content and order has been detected among grass genomes through the use of comparative maps [1,2]. The applications of comparative maps have been discussed many times in the past (See for example: [3]), however genetic maps are not always designed with a comparative study in mind, thus, current maps from different grass species (and in many cases, within the same species) seldom share an adequate number of common (anchor) markers to allow researchers to bridge across maps with an adequate resolution. This is especially true when comparing the genome maps of the Triticeae tribe with the maps of rice or maize, which on average share 3 to 4 markers per wheat homoeologous chromosome group. The lack of anchor markers for bridging across species is exacerbated as new maps are constructed using PCR-based markers such as AFLP, genomic microsatellites and single nucleotide polymorphisms (SNPs) rather than the transferable but laborious cDNA-based RFLP markers.

Genomic SSR (gSSR) markers are biased towards genome specificity [4,5] and generally do not transfer to other species, making them less useful for the generation of comparative maps. For comparative mapping, markers must identify orthologous loci and be polymorphic in two or more species [6].

Recently, several researchers [6-17] have addressed the lack of transferability of gSSRs to other genomes by limiting primer design to transcribed regions, that are expected to have higher levels of conservation across related organisms. Public EST sequence databases from the Poaceae family can be scanned for the presence of SSRs both in protein-coding regions and in untranslated regions of genes (5' or 3' UTRs).

When compared to gSSRs, EST derived SSRs (EST-SSRs) were less polymorphic in a study in hexaploid wheat [9] with only 25% polymorphism, but the successful markers were of high quality and were also polymorphic in durum wheat. Thiel [13] reported a higher level of polymorphism in barley (42%). Lower polymorphism requires more effort to design primers for testing a larger set of candidate markers, but the ease and speed of finding SSRs among freely available EST sequence data offsets this extra effort. This approach is only feasible in species for which there have been EST sequencing projects.

We used the rice genome sequence generated by the International Rice Genome Sequence Project (IRGSP) [18] to identify gSSRs that can potentially serve as sources of markers for mapping. In an equivalent experiment, non-redundant sets of transcript sequences from rice, wheat and barley were scanned (dataset was obtained from the TIGR gene-index databases [19]) and those transcripts containing SSRs were collected and mapped *in-silico *to the rice genome. SSR-containing transcripts derived from different species, sharing a pre-determined threshold of similarity and matching the same location in rice were considered putative orthologs that may be used as anchors in comparative mapping studies.

This paper describes a methodology for developing EST-SSR markers from wheat, barley and rice as markers for developing independent species maps as well as for homologous anchor markers for comparative maps. Over 13,000 untested PCR primer pairs for EST-SSRs were generated from the three gene indices and made available to the research community interested in grass genomes. Researchers are encouraged to evaluate a subset of primer pairs and send feedback regarding their utility to the GrainGenes [20] database for posting. The list (along with all other materials: scripts, programs source code and database schemas) is available from the additional files as well as from the Triticeae EST-SSR Coordination webpage in GrainGenes [21].

## Results

### Frequency of microsatellite types and motifs

Based on combinations of all four nucleotides, the canonical set of SSR motifs is represented by four different duplets (AC, AG, AT, CG), 10 different triplets, 33 different quadruplets and 102 different quintuplet motifs. In the source sequences, all these basic nucleotide motifs can be represented in variant forms of the same basic set or by their reverse complements but to keep a consistency in the database for estimating frequencies, they were transformed into the canonical motifs. Reverse complements and variants would include, for example, CT for AG and GAG for AGG. Sets of unigene sequences such as the TIGR gene indices have the advantage of built-in elimination of redundant SSR counts allowing for more precise estimates of EST-SSR frequency. The rice genomic SSR (gSSR) counts were processed a-posteriori to eliminate redundancy due to BAC/PAC clone overlaps (see methods). Mononucleotide repeats are common in genomic DNA and some are known to be polymorphic but these were deliberately avoided in the unigene database, because they are usually added by the RNA polymerase and are not present in the template DNA (e.g. poly A tails).

Table 1 summarizes the frequencies of SSRs in the TIGR gene indices and the rice genome, grouped by SSR type (di-, tri-, tetra-, and penta-nucleotides) and by several minimum acceptable microsatellite lengths starting at 12 bp or longer. Counts were cumulative, meaning that the totals for SSRs with 12 or more nucleotides included the number of longer SSRs displayed in columns to the right in this table (Table 1). If microsatellite expansion were motif-sequence and location independent, under the null hypothesis one would expect that all types of SSRs would expand at the same rate, and thus, the proportions of SSR types would remain equal from short to long SSRs. However, the proportions of SSR types changed with the length of the SSRs (as did the proportions of motifs, Table 2) and the different SSR types (and motifs) seemed to expand or contract at different rates in the genome of rice, and at different rates when compared to the unigenes of the other two cereals (Tables 1 and 2). SSRs of the trinucleotide type were the most frequent overall. The relatively higher proportion of short trinucleotide-based SSRs in rice unigenes was apparent when compared to the same length categories in wheat or barley unigenes and when compared to the rice gSSRs. The proportion of dinucleotide repeats was greater among genomic microsatellites than among EST-SSRs, and this proportion increased with longer SSRs, overtaking trinucleotide repeats in all datasets when the minimum length was set to 20 bp in gSSRs and 30 bp in EST-SSRs. For instance, in the rice genome 72% of the SSRs longer than 30 bp were of the dinucleotide type; thus, it appears that the rice genomic sequence is relatively richer in dinucleotide SSRs than the gene indices from rice (a subset of the genome) and the other two species.

**Table 1:** Frequency table of perfect and imperfect microsatellites grouped by type of repeat in the non-redundant rice genome (IRGSPnr), rice gene index (Osgi), barley gene index (Hvgi) and wheat's gene index (Tagi) under nine different constraints for minimum SSR length, starting at a minimum of 12 bp. 'n' is SSR count, 'f' is relative proportion (fraction). 'U.f' is unigene fraction: the percentage of unigenes from that species containing at least one SSR.

Dataset	SSRLength	**> = 12**		**> = 14**		**> = 15**		**> = 16**		**> = 18**		**> = 20**		**> = 24**		**> = 30**		**> = 40**	
	SSR type	n	f	n	f	n	f	n	f	n	f	n	f	n	f	n	f	n	f
Osgi	dinucleotide	1751	0.07	1206	0.09	950	0.07	950	0.14	673	0.12	507	0.16	366	0.25	237	0.45	136	0.67
	trinucleotide	17342	0.67	8921	0.63	8921	0.65	4272	0.62	4272	0.75	1993	0.62	912	0.61	242	0.46	56	0.28
	tetranucleotide	4039	0.16	1242	0.09	1242	0.09	1242	0.18	275	0.05	275	0.09	84	0.06	18	0.03	9	0.04
	pentanucleotide	2706	0.10	2706	0.19	2706	0.20	458	0.07	458	0.08	458	0.14	124	0.08	33	0.06	2	0.01
																			
	**Total SSR**	**25838**		**14075**		**13819**		**6922**		**5678**		**3233**		**1486**		**530**		**203**	
	**U.f**		**50%**		**27%**		**27%**		**13%**		**11%**		**6%**		**3%**		**1%**		**0.4%**
																			
Hvgi	dinucleotide	1304	0.07	916	0.10	706	0.08	706	0.15	508	0.15	387	0.17	237	0.25	162	0.48	96	0.67
	trinucleotide	9176	0.52	4326	0.45	4326	0.46	1990	0.43	1990	0.57	971	0.42	464	0.48	115	0.34	34	0.24
	tetranucleotide	4122	0.23	1359	0.14	1359	0.15	1359	0.29	379	0.11	379	0.16	103	0.11	10	0.03	4	0.03
	pentanucleotide	2976	0.17	2976	0.31	2976	0.32	594	0.13	594	0.17	594	0.25	157	0.16	53	0.16	10	0.07
																			
	**Total SSR**	**17578**		**9577**		**9367**		**4649**		**3471**		**2331**		**961**		**340**		**144**	
	**U.f**		**36%**		**20%**		**19%**		**10%**		**7%**		**5%**		**2%**		**1%**		**0.3%**
																			
Tagi	dinucleotide	2606	0.08	1890	0.11	1526	0.09	1526	0.18	1122	0.18	890	0.22	668	0.32	478	0.54	317	0.70
	trinucleotide	18650	0.55	8671	0.50	8671	0.51	3847	0.46	3847	0.61	1840	0.45	989	0.48	343	0.39	124	0.27
	tetranucleotide	8189	0.24	2253	0.13	2253	0.13	2253	0.27	572	0.09	572	0.14	181	0.09	26	0.03	8	0.02
	pentanucleotide	4570	0.13	4570	0.26	4570	0.27	773	0.09	773	0.12	773	0.19	243	0.12	33	0.04	5	0.01
																			
	**Total SSR**	**34015**		**17384**		**17020**		**8399**		**6314**		**4075**		**2081**		**880**		**454**	
	**U.f**		**31%**		**16%**		**16%**		**8%**		**6%**		**4%**		**2%**		**1%**		**0.4%**
																			
IRGSPnr	dinucleotide	32202	0.17	23056	0.21	18541	0.17	18541	0.33	14081	0.34	11286	0.39	8516	0.53	6307	0.72	4284	0.81
	trinucleotide	78832	0.41	38759	0.35	38759	0.36	17615	0.31	17615	0.43	8392	0.29	4431	0.28	1400	0.16	582	0.11
	tetranucleotide	48836	0.25	15030	0.14	15030	0.14	15030	0.26	3789	0.09	3789	0.13	1636	0.10	625	0.07	378	0.07
	pentanucleotide	34102	0.18	34102	0.31	34102	0.32	5591	0.10	5591	0.14	5591	0.19	1401	0.09	385	0.04	54	0.01
																			
	**Total SSR**	**193972**		**110947**		**106432**		**56777**		**41076**		**29058**		**15984**		**8717**		**5298**	

**Table 2:** Frequency table (counts and relative proportions) of the ten most common motifs from perfect and imperfect microsatellites under the same minimum length constraints used in Table 1 found in the non-redundant rice genome (IRGSPnr), rice gene index (Osgi), barley gene index (Hvgi) and wheat's gene index (Tagi).

	**IRGSPnr**			**Osgi**			**Hvgi**			**Tagi**		
**SSRLength**	motif	prop.	count	motif	prop.	count	motif	prop.	count	motif	prop.	count

**> = 12**	CCG	0.18	34238	CCG	0.32	8309	CCG	0.19	3415	CCG	0.20	6643
	AG	0.08	14603	AGG	0.10	2501	AGG	0.09	1536	AGG	0.08	2720
	AT	0.06	11594	ACG	0.06	1677	AGC	0.07	1250	AGC	0.07	2506
	AGG	0.05	10534	AGC	0.06	1583	AAG	0.04	730	AAC	0.05	1805
	ACG	0.04	7151	AG	0.04	1074	AG	0.04	714	AG	0.04	1463
	AGC	0.03	6576	ACC	0.04	1031	ACG	0.04	668	AAG	0.04	1305
	AAG	0.03	4889	AAG	0.04	994	ACC	0.03	604	ACC	0.03	1184
	AAAAG	0.02	4809	ATC	0.02	604	ATC	0.03	446	ACG	0.03	1162
	AAAT	0.02	4756	ATCG	0.01	341	AGGGG	0.02	426	ATC	0.02	758
	ACC	0.02	4517	AAAG	0.01	270	AGGG	0.02	391	AC	0.02	749

**> = 14**	CCG	0.16	17881	CCG	0.33	4622	CCG	0.18	1717	CCG	0.19	3270
	AG	0.09	10293	AGG	0.09	1277	AGG	0.07	716	AGG	0.07	1256
	AT	0.08	9385	AG	0.06	822	AGC	0.06	607	AGC	0.07	1190
	AGG	0.05	5343	ACG	0.06	776	AG	0.06	543	AG	0.07	1140
	AAAAG	0.04	4809	AGC	0.05	774	AGGGG	0.04	426	AAC	0.05	907
	AAAAT	0.03	3221	AAG	0.03	482	AAG	0.04	359	AAG	0.03	559
	ACG	0.03	3178	ACC	0.03	478	ACG	0.03	287	AC	0.03	530
	AGC	0.03	2995	ATC	0.02	273	ACC	0.02	236	ACG	0.03	479
	AGAGG	0.02	2627	CCGCG	0.02	249	AC	0.02	231	ACC	0.03	478
	AAG	0.02	2448	AGAGG	0.02	248	CCCCG	0.02	224	ATC	0.02	322

**> = 16**	AT	0.15	8334	CCG	0.32	2238	CCG	0.17	784	CCG	0.17	1452
	AG	0.14	7931	AG	0.10	674	AG	0.09	424	AG	0.11	951
	CCG	0.14	7820	AGG	0.09	643	AGC	0.07	317	AGC	0.07	560
	AGG	0.04	2438	AGC	0.05	354	AGG	0.07	316	AGG	0.06	542
	AC	0.03	1714	ACG	0.05	339	AAG	0.04	186	AC	0.05	413
	AAAG	0.03	1457	AAG	0.04	249	AC	0.04	175	AAC	0.04	344
	AGC	0.03	1421	ACC	0.03	218	AGGG	0.03	154	AAG	0.03	291
	AAT	0.02	1403	AT	0.02	127	AGGGG	0.03	150	ACG	0.03	221
	AAAT	0.02	1390	ATC	0.02	114	ACC	0.02	115	AGGG	0.02	203
	ACG	0.02	1345	ATCG	0.02	112	ACG	0.02	110	ACC	0.02	202

**> = 18**	CCG	0.19	7820	CCG	0.39	2238	CCG	0.23	784	CCG	0.23	1452
	AT	0.17	7103	AGG	0.11	643	AG	0.09	318	AG	0.12	753
	AG	0.14	5580	AG	0.09	503	AGC	0.09	317	AGC	0.09	560
	AGG	0.06	2438	AGC	0.06	354	AGG	0.09	316	AGG	0.09	542
	AGC	0.03	1421	ACG	0.06	339	AAG	0.05	186	AAC	0.05	344
	AAT	0.03	1403	AAG	0.04	249	AGGGG	0.04	150	AAG	0.05	291
	ACG	0.03	1345	ACC	0.04	218	AC	0.04	124	AC	0.04	267
	AAG	0.03	1228	ATC	0.02	114	ACC	0.03	115	ACG	0.04	221
	AC	0.03	1133	AT	0.02	100	ACG	0.03	110	ACC	0.03	202
	AAAAG	0.03	1131	AGAGG	0.01	66	ATC	0.02	83	ATC	0.02	145

**> = 20**	AT	0.22	6360	CCG	0.32	1032	CCG	0.16	383	CCG	0.15	625
	AG	0.14	4055	AG	0.12	385	AG	0.11	251	AG	0.15	618
	CCG	0.12	3431	AGG	0.10	317	AGC	0.07	170	AGG	0.06	262
	AGG	0.04	1190	AGC	0.05	156	AGGGG	0.06	150	AGC	0.06	250
	AAAAG	0.04	1131	ACG	0.04	143	AGG	0.06	139	AAC	0.06	249
	AAT	0.04	1121	AAG	0.04	134	AAG	0.04	101	AC	0.05	195
	AC	0.03	806	ACC	0.03	97	AC	0.04	85	AAG	0.04	176
	AGAT	0.02	693	AT	0.03	84	ACC	0.03	64	ACC	0.02	92
	AAG	0.02	685	AGAGG	0.02	66	CCCCG	0.03	59	ACGAT	0.02	91
	AGC	0.02	573	ATC	0.02	57	AGAGG	0.02	52	ACG	0.02	77

**> = 24**	AT	0.34	5501	CCG	0.29	436	CCG	0.19	180	AG	0.23	484
	AG	0.16	2504	AG	0.19	278	AG	0.18	171	CCG	0.13	275
	CCG	0.10	1574	AGG	0.10	152	AGC	0.10	93	AAC	0.10	203
	AAT	0.05	866	AAG	0.06	83	AGGGG	0.06	56	AGG	0.06	131
	AGG	0.04	615	AGC	0.05	69	AAG	0.05	48	AC	0.06	127
	AC	0.03	487	ACG	0.04	65	AGG	0.05	47	AAG	0.06	122
	AGAT	0.03	486	AT	0.04	65	AC	0.04	41	AGC	0.06	118
	AAG	0.03	447	ACC	0.03	48	ACC	0.04	37	ACGAT	0.04	86
	AAAAG	0.02	345	ATC	0.02	26	AT	0.03	25	AT	0.03	56
	ACAT	0.02	302	AC	0.01	21	ATC	0.02	21	ATC	0.02	39

**> = 30**	AT	0.54	4680	AG	0.35	184	AG	0.39	133	AG	0.42	368
	AG	0.15	1343	CCG	0.17	90	AGGGG	0.09	29	AAC	0.15	132
	AAT	0.05	420	AT	0.09	46	CCG	0.07	24	AC	0.08	68
	CCG	0.04	310	AGG	0.08	40	AAG	0.07	23	AAG	0.07	59
	AGAT	0.03	295	AAG	0.06	32	AGC	0.07	23	AT	0.05	42
	AC	0.03	280	AGC	0.05	24	AC	0.05	16	CCG	0.05	42
	AAG	0.03	236	ACG	0.03	16	AT	0.04	13	AGC	0.04	37
	ACAT	0.02	198	ACC	0.02	13	AAC	0.03	10	AGG	0.03	27
	AGG	0.02	176	AAC	0.02	10	ACC	0.03	9	AAT	0.01	13
	AAAAG	0.01	119	AGAGG	0.02	9	CCCCG	0.02	8	ATC	0.01	12

**> = 40**	AT	0.69	3631	AG	0.51	104	AG	0.60	86	AG	0.57	260
	AG	0.09	494	AT	0.14	29	AGC	0.07	10	AAC	0.14	62
	AAT	0.07	353	AAG	0.08	17	AAG	0.06	9	AC	0.07	31
	AGAT	0.04	192	AGC	0.04	8	AT	0.06	8	AAG	0.07	31
	AC	0.03	157	CCG	0.03	7	AGGGG	0.05	7	AT	0.06	26
	ACAT	0.03	153	ACG	0.03	6	AAC	0.03	4	AGC	0.03	14
	AAG	0.02	109	AGG	0.03	6	ACC	0.01	2	ACAT	0.01	5
	CCG	0.01	27	AAC	0.02	5	ACAT	0.01	2	AGG	0.01	3
	AAC	0.00	24	ATC	0.02	4	ATC	0.01	2	CCG	0.01	3
	AGG	0.00	19	ACAT	0.02	4	ACT	0.01	2	AAT	0.01	3

### ESTs are a rich source of SSRs

The abundance of SSRs (perfect and imperfect) in the unigenes can range from one in every 100 to one in every two unigenes depending on the minimum length (Table 1). When all SSRs with a minimum length of 12 bp are tabulated, 50%, 36% and 31% of rice, barley and wheat unigenes have at least one SSR. When the minimum length was raised to 16 bp the proportion was reduced to 13, 10 and 8%, respectively. Rice unigenes had a higher frequency of SSRs than did barley and wheat for most minimum lengths, but not for SSRs longer than 20 bp, where the relative abundance was similar in all three gene indices. The nearly two-fold difference at a minimum length of 18 bp was mostly due to the high abundance of trinucleotide SSRs in rice unigenes relative to wheat and barley. The abundance of trinucleotide repeats decreased by about one half for each repeat unit added to the series. The decline in abundance was steeper for tetranucleotide and pentanucleotide repeats but was less than one half for dinucleotide repeats, which at lengths greater than or equal to 30 bp, became the predominant type in all datasets. At 30 bp or longer, the AT motif was most common among gSSRs while AG was more numerous among EST-SSRs motifs (Table 2).

Wheat unigenes contained a larger number of SSRs for all repeat length categories, followed by rice and barley unigenes. This is probably because wheat has more than twice the number of unigenes than rice or barley with 109,782 for wheat, 51,569 for rice and 48,159 for barley. The larger number of unigenes in hexaploid wheat may result from divergence of the genes in the three genomes, but also from a relatively larger EST dataset, i.e., more ESTs have been sequenced for wheat, with a sequence redundancy of 3.8×, versus 6× and 2.7× for barley and rice, respectively (see methods).

The number of the ten most frequent motifs was tabulated for different minimal SSR lengths (Table 2). The relative proportions of motifs fluctuated with different length constraints as well as source species. At a minimum SSR length of 12 bp, CCG was predominant in all datasets, but AT and AG were more frequent in the higher range of minimal SSR lengths. Among dinucleotides in the rice EST-SSRs, AG and AT were the most common, but AG and AC were more common in wheat and barley EST-SSRs. Besides CCG, other frequent trinucleotide motifs were AGG and AGC. The trinucleotide (CCG)n microsatellite was present in both coding regions and UTRs. In coding regions, this triplet has the potential to code for the amino acids proline (CCG), arginine (CGG), alanine (GCC), glycine (GGC), but among these, expansion of the motif leading to additions of the amino acid proline could have the strongest effects on protein structure while alanine and glycine would have relatively small effects.

The longest SSRs were genomic microsatellites (as long as 726 bp). Unigenes had few SSRs longer than 40 bp, up to 333 bp in wheat ESTs. Often these were not useful for developing SSR markers because no flanking sequence was available to design primers. The overall mean length for rice gSSRs equal to or longer than 12 bp was 16.5 (s.d = 12.7), with no significant differences in mean gSSR lengths among the chromosomes in rice, while the mean length for EST-SSRs was 15.3 (s.d = 6) with no significant differences among the three species gene indices (t-test 2-sample with unequal variances, *p *> 0.01).

### Density of gSSRs and comparison to EST-SSRs mapped *in silico *in rice chromosome 1

The contiguity of the pseudomolecule sequence of rice chromosome 1 (R1) (a virtual sequence composed of the assembly of tiling path clones), with only eight gaps for the whole chromosome, provided a convenient framework of coordinates for calculating density estimates of gSSRs features (by *in-silico *scanning with the Sputnik program), and for anchoring rice, wheat and barley unigenes associated with microsatellites (by sequence similarity). Best similarity matches between the rice genome and 8,259 barley, 16,917 rice and 13,565 wheat EST-SSR unigenes were identified using BLASTN. From these, a total of 6,373 EST-SSRs mapped to R1 pseudomolecules including 1,104 from barley, 3,568 from rice and 1,701 from wheat with 88.8%, 98.4% and 89.1% average sequence similarity respectively.

**Figure 1:**
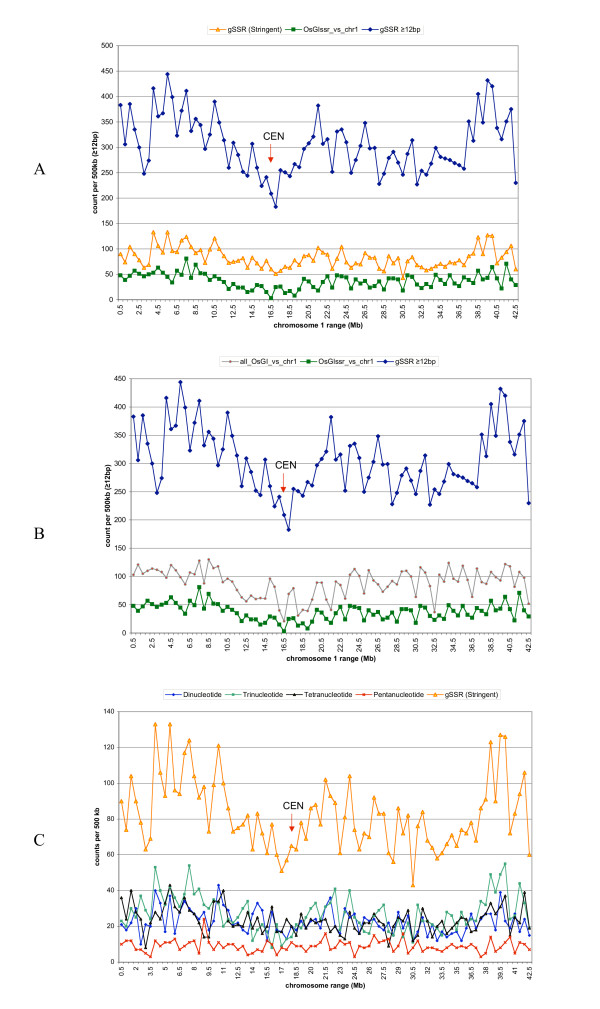
A) Features in the contiguous 42.5 Mb of rice chromosome 1: Comparison between the density (counts per 500 kbp) of the stringent subset of gSSRs (gSSR Stringent: ≥ 16 bp tetranucleotides, ≥ 18 bp dinucleotides and trinucleotides, and ≥ 20 bp pentanucleotides; in orange), density of the best matches to rice unigenes associated with microsatellites (OsgiSSR_vs_chr1; in green) and density of genomic microsatellites (gSSR; in blue) with length ≥ 12 bp. The centromere location is indicated by "CEN". B) Features in the contiguous 42.5 Mb of rice chromosome 1: Comparison between the density of gSSRs ≥ 12 bp (in blue), the density of best sequence similarity matches to rice unigenes associated with microsatellites (OsgiSSR_vs_chr1; in green), and the density of best sequence similarity matches between rice unigenes (Osgi_vs_chr1) and rice chromosome 1 (gray line and red points). C) Rice chromosome 1 density plots of the stringent subset of gSSRs and its component types (di-, tri-, tetra- and pentanucleotides)

Density of gSSRs equal to or longer than 12 bp in R1 ranged from 1 gSSR in 2.8 kbp near the centromere to 1 gSSR per 1.1 kbp in the distal regions (Figure 1A). For a more stringent subset of gSSRs (≥ 16 bp tetranucleotides, ≥ 18 bp dinucleotides and trinucleotides, and ≥ 20 bp pentanucleotides), the density ranged from 1 gSSR in 10 kbp around the centromere to 1 gSSR in 3.8 kbp in the densest region of the short arm (Figure 1A).

**Figure 2:**
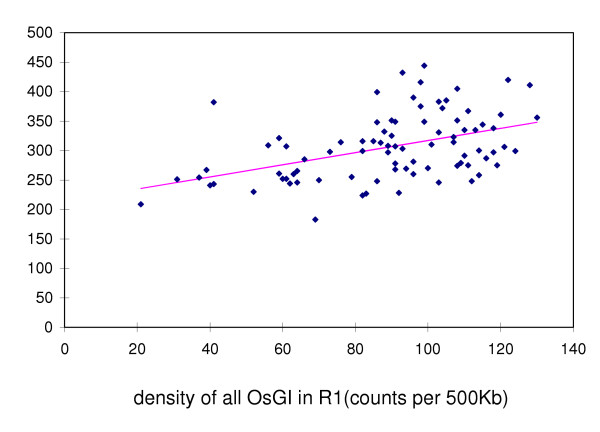
Linear regression of the density of genes in rice chromosome 1 (roughly estimated by the matches of OsGI sequences to this chromosome: Osgi_vs_chr1) on the density of gSSRs (≥ 12 bp) in rice chr1. Pearson correlation is 0.45. Regression coefficients are highly significant (*P*-value = 1.2E-05). Density is expressed in counts per 500 kbp.

The comparison of the density of gSSRs to, a) the density of all rice unigenes mapped to R1, and b) the density of EST-SSRs (a subset from the unigenes) mapped to R1 provided an estimate of the relationship between gSSRs and gene regions in the rice genome. There was a striking resemblance in the patterns of the plots for the density of gSSRs and the density of unigene-derived EST-SSRs in R1 pseudomolecules (Figure 1A). The similarity in density patterns was less apparent but still present between gSSRs and R1 matches to all rice unigenes (Figure 1B), and these densities were significantly correlated (*r *= 0.45, *p *≤ 1E-5; Figure 2).

A decomposition of the set of stringent gSSRs (see the methods section for criteria defining the "stringent gSSRs") by types in R1 (Figure 1C) showed that the relative proportions of the pentanucleotide gSSRs ≥ 20 bp were consistently lower over the majority of the chromosome, while the proportion of the other three types of microsatellites was higher, indicating that pentanucleotides are only a small component in the non-homogeneous distribution of the stringent subset.

### Development of primers for cereal EST-SSRs

We designed primer pairs for 5,425 wheat, 3,036 barley and 4,726 rice EST-SSRs conforming to the stringent restrictions described in the methods. The average product size expected from the set of designed primers was 217 bp for rice EST-SSRs, 213 for wheat and 218.9 for barley.

Of those EST-SSRs, 42% of the wheat and 56% of the barley were mapped *in-silico *to the rice genome. The additional file 1 contains the list of primer pairs that can be downloaded for testing.

## Discussion

### Are microsatellites preferentially associated with gene-rich DNA in rice?

Morgante and colleagues [22] reported that in plants, gSSRs were preferentially associated with non-repetitive DNA such as the gene-rich regions. They found a highly significant, positive, linear relationship (*r*^2 ^= 0.94, *p *< 0.006) between genomic microsatellite frequency and the percentage of single copy DNA in several plant species with a wide range of genome sizes. Estimates of repetitive and non-repetitive single-copy DNA fractions were based on reviews of the literature describing renaturation kinetics experiments for each of the species. Plant species that have gone through genome expansion due to retrotransposon amplification, such as maize and wheat, had a lower genomic microsatellite frequency indicating that SSR frequency is not a function of overall genome size but rather the relative proportion of single-copy DNA.

In this study, the best similarity matches between rice unigene sequences and the genomic sequence of rice chromosome 1 were used to estimate the density of transcribed regions along R1. This estimate was compared to both the density of gSSRs and the density of EST-SSRs (the latter group being the intersection of the set of gSSRs and the set of transcribed regions, or unigenes). The density pattern of transcribed regions (unigenes in R1) and of SSRs within transcribed regions (unigenes in R1 with SSRs or EST-SSRs) followed closely the density pattern of gSSRs in rice chromosome 1 (*r *= 0.45, *p *< 1 × 10^-5 ^and *r *= 0.62, *p *< 1 × 10^-10^, respectively) (Figures 1A, 1B and 2).

The density (counts per 500 Kbp) of gSSRs along R1 was higher than both the density of transcribed regions and the density of EST-SSRs. A large number of gSSRs that are not already included in the set of EST-SSRs could still be associated with genes because, as Figure 1B suggests, they are preferentially found in genic regions near promoter regions or inside introns and away from the highly repetitive and gene-poor DNA in heterochromatin. In rice, (AT)n SSRs are rare among ESTs, but are the most common gSSRs among the long group (= 20 bp). The (AT)n SSR motif is frequently found along with sequences of the Micropon family of MITEs [23,24] which are associated with gene-rich regions.

Other reports have documented a role for SSRs that are associated with genes in the control of gene expression. For example, several human diseases have been linked with events of triplet expansions in the past [25]. Chromatin remodeling and gene silencing via histone-deacetylation/cytosine-methylation are among the putative functions of SSRs in the vicinity of genes, especially if GC rich. Coffee [26] showed that histone deacetylation (and methylation of CpG bases) leading to lower expression at the FMR1 locus in fragile X was a consequence of CCG repeat expansion. In another example, the expansion of a (AGC)n SSR in the 3' UTR of the myotonic dystrophy (DM) protein kinase gene could potentially affect the expression due to changes in local chromatin structure [27]. It has been found that DM patients have a reduced or complete loss of a nuclease-hypersensitive site in the region of the gene. Further analysis showed that the majority of DM protein kinase transcripts from cells carrying the repeat expansion also lacked the last two exons of a normal transcript, showing that the repeat expansions affected the splicing at the 3' end.

In rice, although the presence of a single-base mutation breaking an intron splice site is more directly responsible for the difference in phenotypes of the waxy gene, polymorphism due to SSR expansion has been associated with variation of expression levels in different japonica and indica varieties [28,29]. The effect that microsatellites might have in gene expression in plants may be observed as natural phenotypic variation.

### A strategy to exploit the EST database for microsatellite markers

One strategy to better exploit a database of EST-SSRs in order to find polymorphic markers is to first sample the longest SSRs (≥ 30 bp), favoring dinucleotide repeats, then follow with trinucleotide, tetranucleotide and pentanucleotide repeats [23]. After exhausting the longest SSRs, one would then proceed with another cycle to select shorter SSRs. Short trinucleotide-based microsatellites such as (CCG)n, the most abundant group overall (Table 2), are more likely to derive from coding regions, thus reducing the chances for finding polymorphism [13]. This strategy is based on the following observations from our results and the literature: 1) Dinucleotides are a better source of polymorphic markers than the other types [30]. 2) Longer SSRs generally have a higher tendency to be polymorphic [23]. 3) SSRs deriving from UTRs have the potential for a higher polymorphism than those derived from coding regions, which are constrained by purifying selection [30].

One percent of unigenes from the three species examined in this study have SSRs starting with a minimum of 30 bp (Table 1). Overall, 880 wheat unigenes, 530 rice unigenes and 340 barley unigenes contain at least one of these long microsatellites and primer pairs were successfully designed for 451, 276 and 148 of these long EST-SSRs in wheat, rice and barley, respectively. At this minimum length, trinucleotide repeats were not the most frequent. Nearly 50% of the EST-SSRs longer than 30 bp were based on dinucleotide repeats (Table 1), with (AG)n being the most common motif (Table 2). Yet, the frequency of SSR types among those for which primers could be designed did not follow this pattern. Trinucleotide repeats were still the most common type in this group, followed by dinucleotides. This was due to the fact that dinucleotides are found preferentially in the UTRs of transcripts, and their sequences had fewer surrounding bases to anchor acceptable primers.

After relaxing the microsatellite length constraint to a minimum of 20 bp, the overall number of SSRs in the unigenes increased to around 5%. An additional 3,195, 2,703 and 1,991 EST-SSRs in wheat, rice and barley become available for primer design. Acceptable primer pairs were designed for 2,622 wheat, 2,183 rice and 1,476 barley EST-SSRs in this category (which included the set mentioned previously).

The set of EST-SSRs with acceptable primer pairs (Additional file 1) were selected from among all dinucleotide and trinucleotide EST-SSRs with a minimum of 18 bp, tetranucleotide EST-SSRs longer than 16 bp and pentanucleotide EST-SSRs 20 bp or longer. However, from 5,424 wheat unigenes associated with microsatellites and having a set of PCR primers in our database, only 2,323 had a best match in the rice BAC/PACs with our stringency settings. The rest (57%) are not anchored to the rice genome but still have potential to provide polymorphic wheat microsatellite markers. The same applies to 44% of the barley EST-SSRs with acceptable primers. The reasons for a large number of wheat/barley unigenes without matches to rice genomic sequence include not having the complete sequence of the rice genome available (the majority of clones were still in sequencing phase 2, with gaps) and having a relatively high stringency setting for filtering wheat and barley sequence comparisons to rice. In previous comparisons between wheat EST unigenes and the same version of the rice genome sequence draft [31,32], we found that 40% of the unigenes did not significantly match a sequence in the rice genome.

## Conclusion

The relative proportions of di-, tri-, tetra-, and penta nucleotide repeats and motifs varied widely depending on length and were not consistent among the species examined. We have shown that ESTs are a good source of SSRs that can be exploited to develop microsatellite markers for wheat, barley and rice. The advantage to this approach is that the sequences are already available resulting in a lower cost than designing and testing microsatellites from anonymous genomic libraries, even if the polymorphism rate for EST-derived markers is lower.

EST-SSRs are useful for enhancing individual species maps, but can be used as anchor probes for creating links between maps in comparative studies when designed from sets of orthologous genes, as demonstrated by Yu et al [33]. The annotation and/or the sequence similarity between putative orthologous genes from two related species can provide the basis for their use in comparative maps. More than 13,000 primer pairs were designed to amplify fragments from a stringent subset of EST-SSRs in wheat, rice and barley and are available to the public for testing.

Using a different methodology, our results substantiated the report by Morgante et al [22] suggesting that microsatellites are predominantly found in the vicinity of genes. In some instances, their presence in the vicinity of genes may implicate a regulating function by mechanisms involving chromatin remodelling and DNA methylation.

## Methods

### Source of unigene sequences

TIGR's non-redundant gene indices [34] from wheat, barley and rice were downloaded in January of 2003. The databases were: OsGi rel.11 (January 2003) for rice transcripts, with 51,569 non-redundant sequences after processing 139,918 ESTs; HvGi rel. 5 (December 2002) for barley transcripts, with 48,159 non-redundant sequences from 304,061 initial sequences and TaGi rel. 6 (January 2003) for wheat transcripts with 109,782 non-redundant sequences (415,125 initial sequences).

### Source of rice genomic sequences

All analyses of the rice genome used the version released in December 2002 by the International Rice Genome Sequencing Consortium [18] and consisted of a minimum tiling path of 3,280 BAC or PAC clones for the 12 rice chromosomes. There were nine pseudomolecules of assembled, contiguous sequence available for rice chromosome 1 that replaced the overlapping clones in that chromosome [35]. For the rest of the genome, accession numbers for the individual BAC/PAC clones in the tiling path were used to download the corresponding sequence from NCBI GenBank [36]. The tiling path for chromosomes 2 to 12 was used to facilitate the posterior ordering of clones.

### Scanning of the rice genome and the non-redundant EST-datasets for SSRs

The TIGR gene indices and the genome of rice were scanned with a modified version of Sputnik [22] available from the University of Delaware [37] to find all perfect and imperfect SSRs having 2 to 5 nucleotides in the basic repeat unit and at least 12 bp in total length. For imperfect SSRs, up to 10% sequence deviation from a perfect SSR was included. We modified the way the program handles input sequences in the NCBI FASTA format and the format of the program's output, making it easier to export to relational databases. No changes were made to the underlying algorithms written by C. Abajian and modified by Morgante's group. The version of Sputnik used to generate the microsatellite data for this report can be obtained from the GrainGenes EST-SSR coordination webpage [21], or by downloading the additional file 2.

In order to eliminate the problem of counting the same microsatellites several times in the rice genome due to the redundancy created by overlapping regions between contiguous BAC/PACs in chromosomes 2 to 12, the gSSRs were annotated as redundant or not, according to their location in the tiling path. When located to a region in the BAC/PAC that overlapped with a neighbor clone (based on the tiling path information as well as MegaBLAST [38] pairwise alignments) only the SSRs belonging to the overlapped region of the top (northern) clone were counted while those present in the overlapped region of the bottom (south) clone were ignored. Of course, all SSRs found in unique, non-overlapping regions of rice clones were counted. A perl script that performed queries and updates to the SQL database (via the DBI perl module) scanned the tables of genomic microsatellites and flagged them according to the procedure explained above. Thus 24% of the genomic microsatellites (of length ≥ 12 bp) found in the rice BAC/PAC clones were ignored, as they were duplicates due to clone overlaps.

Table 1 shows the counts and relative proportions of SSRs found in the four datasets (rice, wheat and barley gene indices as well as in the non-redundant rice genomic) for dinucleotides, trinucleotides, tetranucleotides and pentanucleotides when having different minimum microsatellite lengths (greater than or equal to 12 bp) as the starting point. Table 2, on the other hand, shows the relative proportions of the ten most common motifs for each dataset when subject to different constraints for minimum microsatellite lengths.

### *In-silico *mapping of grass Non-redundant EST-SSRs

The set of EST unigenes associated with SSRs from wheat and barley was matched against the sequence of the rice genome to provide a putative map location in rice. Only the best hits were recorded for any given EST unigene. The similarity threshold was set at an E-value < 1 × 10^-10 ^and at least 80% similarity over 100 bp of minimum alignment. Rice EST unigenes were matched with the same criteria except for a higher similarity threshold of 95%. The inferred location in the rice genome for rice EST unigenes was used to estimate the proportion of rice gSSRs that were associated with regions containing genes.

### PCR primer design

We developed a perl script (see Additional file 3) that automatically queries the database of EST-SSRs to design primers in batch based on what was learned in previous experiments and on recommendations found in the literature to maximize the chance of selecting polymorphic microsatellite markers. The script used the BioPerl module [39] to control the Primer3 core program [40,41], feeding each of the SSR source sequences and specifying the target regions to be amplified via PCR.

EST-SSRs were selected for primer design when conforming to the following more stringent restrictions (referred to as the set of stringent SSRs or gSSR stringent):

a) The SSRs are dinucleotides or trinucleotides of length equal or larger than 18 bp, tetranucleotides equal or larger than 16 bp or pentanucleotides equal or larger than 20 bp.

b) The imperfect SSRs have less than 10% mismatches or gaps relative to a perfect SSR of the same length and motif.

c) There is a minimum of 50 bp surrounding the SSR edges in the source sequence to allow for possible primer design.

The parameters used for the Primer3 program specified an optimal Tm of 60°C with a minimum and maximum of 57°C and 65°C, respectively, and a 30% to 70% GC content with a low chance of dimer or hair-loop formation. The range for PCR product length was set to be between 100 and 300 bp.

## Abbreviations

EST: Expressed Sequence Tag. SSR: Simple Sequence Repeat. gSSR: genomic SSR. EST-SSR: EST-derived SSR. UTR: Untranslated region flanking a coding region in DNA and messenger RNA. IRGSP: International Rice Genome Sequence Project. R1: rice chromosome 1. BAC/PAC: Bacterial artificial chromosomes or bacteriophage P1 artificial chromosomes (for cloning of large DNA fragments). MITE: Miniature Inverted-repeat Transposable Element.

## Authors' contributions

ML did all programming and design of computational experiments and databases. RVK contributed in the first database design. JY did wet-lab testing of a subset of primer pairs.

ML and MES drafted the manuscript. All authors read and approved the final manuscript.

## Supplementary Material

Additional File 1 **EST-SSR designed primers **Table listing the stringent subset of SSRs (≥ 16 bp tetranucleotides, ≥ 18 bp dinucleotides and trinucleotides, and ≥ 20 bp pentanucleotides) found in rice, barley and wheat gene indices for which primer sequences were designed, the source sequences, the primers, their *in-silico *mapping in the rice genome (to BAC/PAC clones or pseudo-molecule) and relevant metadata. File is a spreadsheet table, compressed with the zip program. The file (14 Mb uncompressed) is also available at the GrainGenes Triticeae EST-SSR Coordination page Click here for file

Additional File 2 **Modified Sputnik source code and executable **This is the source code with modifications, to the microsatellite searching program "Sputnik", originally written by Chris Abajian from the University of Washington at Seattle. The set of files are compressed using the zip program. File is also available at Click here for file

Additional File 3 **Perl script to design primers in batch **The script uses the Bioperl perl modules to control the Primer3 program in order to design primers from a microsatellite database stored in a MySQL database. The script can be modified to accommodate similar schemas on any database engine supported by the perl DBI module. File is also available at Click here for file
