# Supplementary material for: Nonrandom distribution and frequencies of genomic and EST-derived microsatellite markers in rice, wheat, and barley
Source: BMC Genomics. 2005 Feb 18;6:23. doi: 10.1186/1471-2164-6-23 (PMC550658; doi:10.1186/1471-2164-6-23)
Supplement: Additional File 2 — Modified Sputnik source code and executable This is the source code with modifications, to the microsatellite searching program "Sputnik", originally written by Chris Abajian from the University of Washington at Seattle. The set of files are compressed using the zip program. File is also available at [file 1471-2164-6-23-S2.zip › Cornell_Dist/sputnik/sputnik.plg]

```
# Build Log


### --------------------Configuration: sputnik - Win32 Release--------------------


### Command Lines

Creating temporary file "C:\DOCUME~1\cmlarota\LOCALS~1\Temp\RSP371E.tmp" with contents
[
/nologo /ML /W3 /GX /O2 /D "WIN32" /D "NDEBUG" /D "_CONSOLE" /D "_MBCS" /Fp"Release/sputnik.pch" /YX /Fo"Release/" /Fd"Release/" /FD /c 
"C:\DATA\source\sputnik_source_Mar2003\getOpt.c"
]
Creating command line "cl.exe @C:\DOCUME~1\cmlarota\LOCALS~1\Temp\RSP371E.tmp" 
Creating temporary file "C:\DOCUME~1\cmlarota\LOCALS~1\Temp\RSP371F.tmp" with contents
[
kernel32.lib user32.lib gdi32.lib winspool.lib comdlg32.lib advapi32.lib shell32.lib ole32.lib oleaut32.lib uuid.lib odbc32.lib odbccp32.lib /nologo /subsystem:console /incremental:no /pdb:"Release/sputnik.pdb" /machine:I386 /out:"Release/sputnik.exe" 
.\Release\getOpt.obj
.\Release\sputnik.obj
]
Creating command line "link.exe @C:\DOCUME~1\cmlarota\LOCALS~1\Temp\RSP371F.tmp"

### Output Window

Compiling...
getOpt.c
Linking...

### Results

sputnik.exe - 0 error(s), 0 warning(s)
```
